# Porphyrin-derived carbon dots for an enhanced antiviral activity targeting the CTD of SARS-CoV-2 nucleocapsid

**DOI:** 10.1186/s43141-023-00548-z

**Published:** 2023-10-06

**Authors:** Azzania Fibriani, Audrey Angelina Putri Taharuddin, Nicholas Yamahoki, Rebecca Stephanie, Jessica Laurelia, Dian Fitria Agustiyanti, Popi Hadi Wisnuwardhani, Marissa Angelina, Yana Rubiyana, Ratih Asmana Ningrum, Andri Wardiana, Desriani Desriani, Ferry Iskandar, Fitri Aulia Permatasari, Ernawati Arifin Giri-Rachman

**Affiliations:** 1 School of Life Sciences and Technology, Institut Teknologi Bandung, Bandung, 40132 Indonesia; 2 Research Center for Nanoscience and Nanotechnology, Institut Teknologi Bandung, Bandung, 40132 Indonesia; 3 Research Center for Genetic Engineering, Indonesian National Research and Innovation Agency (BRIN), Cibinong, 16911 Indonesia; 4 Research Center for Pharmaceutical Ingredients and Traditional Medicine, Indonesian National Research and Innovation Agency (BRIN), Serpong, 15314 Indonesia; 5 Department of Physics, Faculty of Mathematics and Natural Sciences, Institut Teknologi Bandung, Bandung, 40132 Indonesia; 6 Collaboration Research Center for Advanced Energy Materials, National Research and Innovation Agency-Institut Teknologi, Bandung, Bandung, 40132 Indonesia; 7 Research Center for Chemistry, National Research and Innovation Agency (BRIN), Serpong, 15314 Indonesia

**Keywords:** Antivirus, Cytotoxicity, Phytochemicals, Porphyrin, Carbon dots, Dimer-based screening system, Nucleocapsid, Coronavirus, SARS-CoV-2, COVID-19

## Abstract

**Background:**

Since effective antiviral drugs for COVID-19 are still limited in number, the exploration of compounds that have antiviral activity against SARS-CoV-2 is in high demand. Porphyrin is potentially developed as a COVID-19 antiviral drug. However, its low solubility in water restricts its clinical application. Reconstruction of porphyrin into carbon dots is expected to possess better solubility and bioavailability as well as lower biotoxicity.

**Methods and results:**

In this study, we investigated the antiviral activity of porphyrin and porphyrin-derived carbon dots against SARS-CoV-2. Through the in silico analysis and assessment using a novel drug screening platform, namely dimer-based screening system, we demonstrated the capability of the antivirus candidates in inhibiting the dimerization of the C-terminal domain of SARS-CoV-2 Nucleocapsid. It was shown that porphyrin-derived carbon dots possessed lower cytotoxicity on Vero E6 cells than porphyrin. Furthermore, we also assessed their antiviral activity on the SARS-CoV-2-infected Vero E6 cells. The transformation of porphyrin into carbon dots substantially augmented its performance in disrupting SARS-CoV-2 propagation in vitro.

**Conclusions:**

Therefore, this study comprehensively demonstrated the potential of porphyrin-derived carbon dots to be developed further as a promisingly safe and effective COVID-19 antiviral drug.

**Supplementary Information:**

The online version contains supplementary material available at 10.1186/s43141-023-00548-z.

## Introduction

It has been 3 years since the coronavirus disease-19 (COVID-19) pandemic was declared in March 2020. Currently, there are many vaccine products available to prevent severe acute respiratory syndrome-coronavirus-2 (SARS-CoV-2) infection. Nine vaccine products have acquired Emergency Use Authorization (EUA) from the US Food and Drug Administration (FDA) as of December 31, 2022 [1]. Still, the effective antiviral drugs to help eliminate the viruses in the patient’s body are limited in number. As of December 31, 2022, there were only three antiviral drugs that have received the FDA EUA for the treatment of COVID-19 patients: molnupiravir, Paxlovid (ritonavir-boosted nirmatrelvir), and remdesivir. The first mentioned is a nucleotide analog that disrupts the virus replication in the host cells, while the other two work by inhibiting the viral enzymes [1, 2]. Therefore, the exploration of new effective antiviral drugs against SARS-CoV-2 is obviously needed.

Indeed, the development of novel drugs is always challenging since it races against time as the outbreak progresses to over [2]. The drug candidates need to pass many assessments before they can be used for humans. In addition, conventional drug screening procedures require high biosafety level laboratories, at least BSL-3 [3, 4]. The limited number of adequate research facilities is a serious bottleneck in the discovery of new drugs, especially for lower-middle-income countries. For those reasons, various alternative high-throughput platforms—using synthetic peptide [5], recombinant virus [6], virus-like particle (VLP) [7], and engineered bacteria [8]—have been developed to screen potential drug candidates in more effective and efficient ways.

**Fig. 1:**
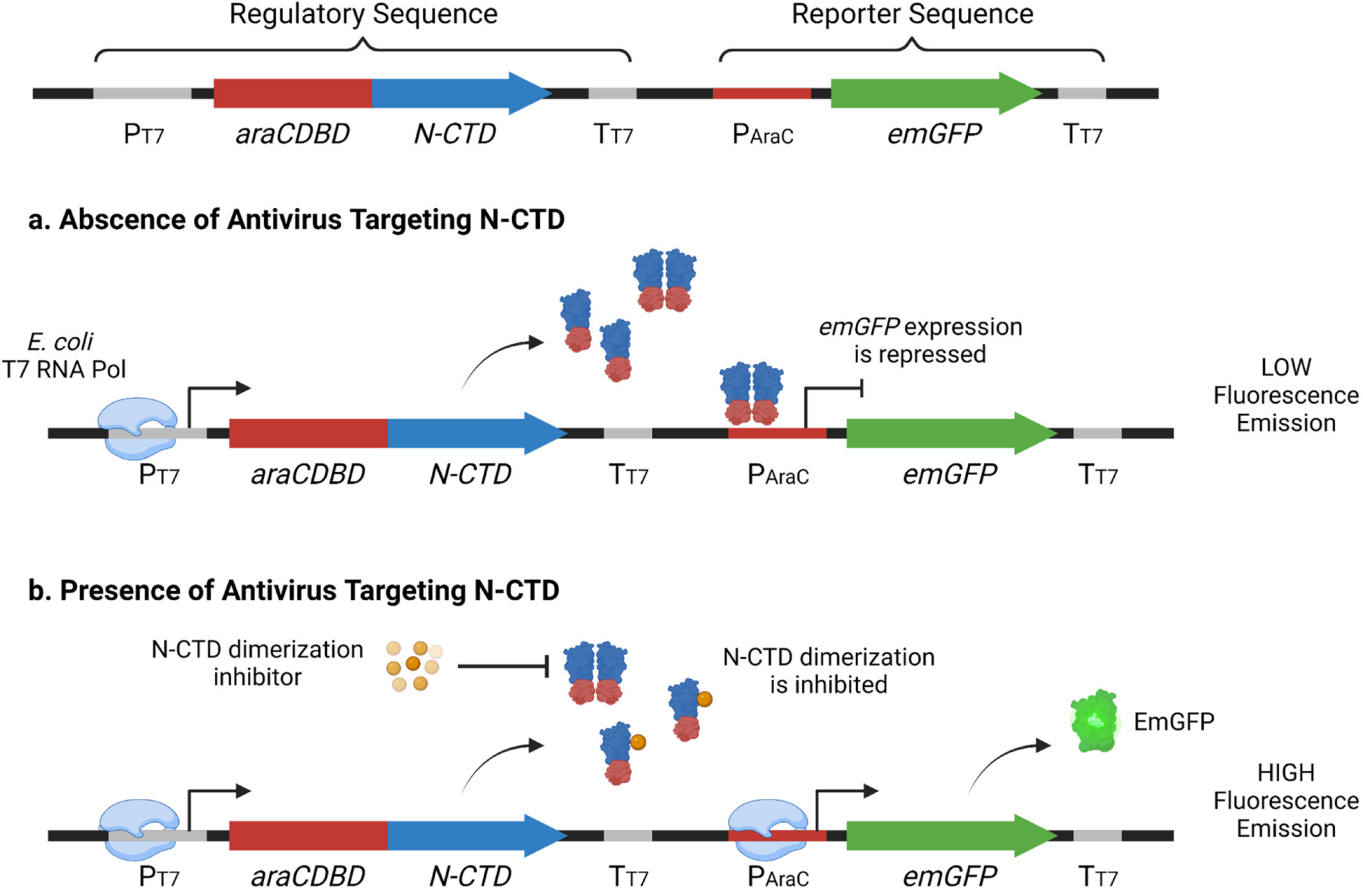
Dimer-based screening system (DBSS) for COVID-19 antiviral drug screening that applied in this study. The system screened the antiviral drug candidates that have the capability to inhibit dimerization of the C-terminal domain (CTD) of SARS-CoV-2 nucleocapsid. In the presence of viral protein inhibitors, the genetically engineered *E. coli* will emit a high fluorescence signal. The illustration was created with BioRender

Using a synthetic biology approach, a novel target-based drug screening platform, called a dimer-based screening system (DBSS), has been developed for identifying antimicrobial drug candidates from diverse bioactive compounds and drug repurposing for various pathogens, such as *Mycobacterium tuberculosis* [9, 10], human immunodeficiency virus (HIV) [11–14], and hepatitis B virus (HBV) [15]. Inspired by Furuta et al. [16] and Okada et al. [17], DBSS assesses the capability of drug candidates to inhibit the dimerization of bacterial or viral proteins by using a genetically engineered *Escherichia coli*. Since the platform did not directly involve the target pathogen, it could be done in low biosafety level laboratories. Adopting the concept, Fibriani et al. [18] developed a DBSS targeting the C-terminal domain (CTD) of the SARS-CoV-2 nucleocapsid protein as a screening system for COVID-19 drug candidates (Fig. 1).

**Fig. 2:**
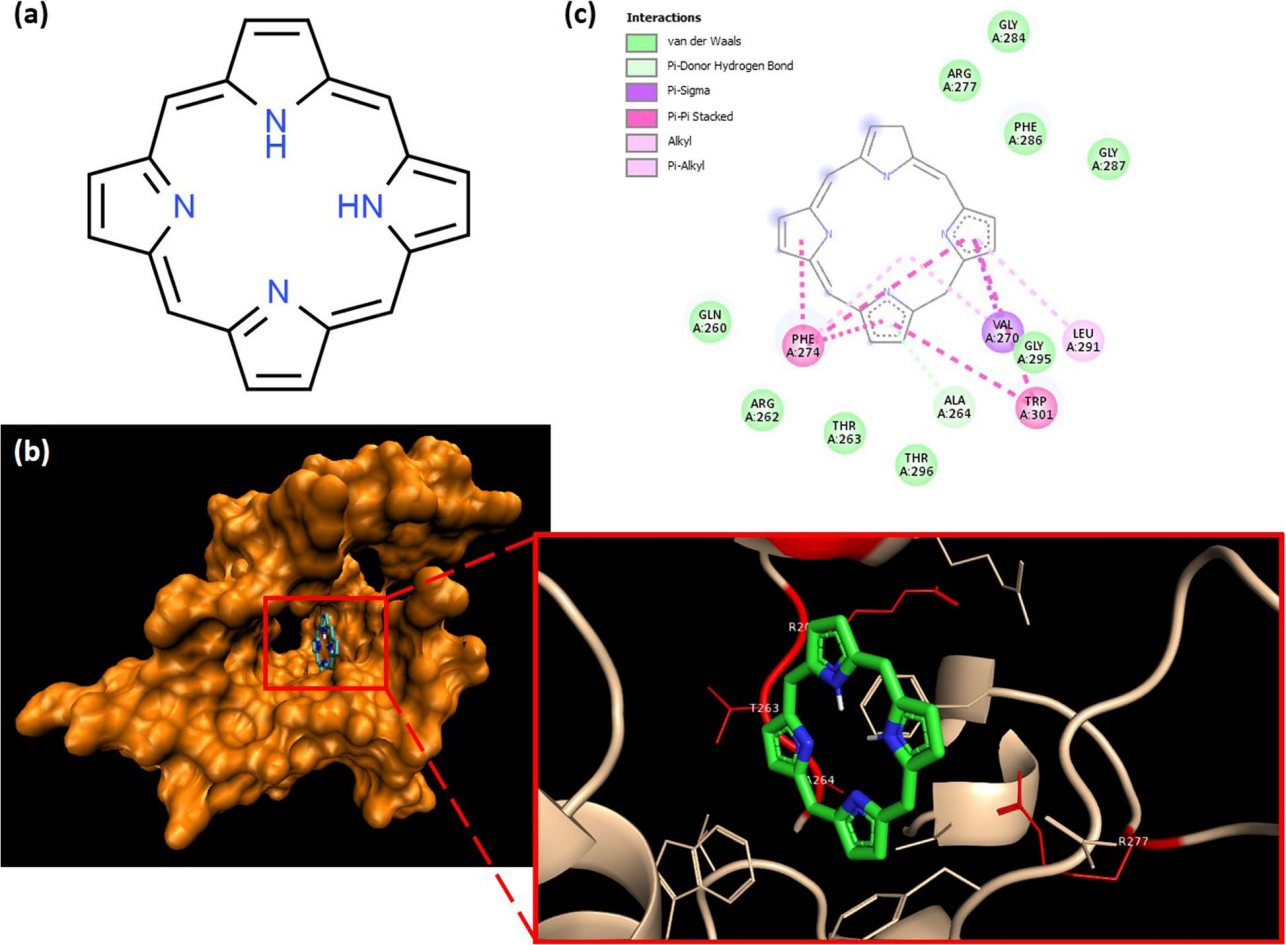
Molecular docking analysis of porphyrin (PubChem ID: 66,868) with the C-terminal domain (CTD) of SARS-CoV-2 nucleocapsid (PDB ID: 6ZCO). Structure of porphyrin (C_20_H_14_N_4_; CAS No. 101–60-0). Porphyrin molecule consists of four pyrrole rings linked by methine bridges (**a**). The binding of porphyrin on the target viral protein was visualized (**b**). Furthermore, the residues of the target viral protein that interacted with the ligand were defined (**c**). The visualization of docking results was prepared using BIOVIA Discovery Studio

Porphyrin is one of the natural bioactive compounds that has demonstrated antiviral activities against numerous enveloped viruses, including HBV, HIV, dengue virus, Lassa virus, and also influenza A virus [19, 20]. Porphyrin is a group of organic compounds with an aromatic heterocyclic macrocycle structure (Fig. 2a). It could be easily found in nature and plays essential roles in many organisms. Due to its structure, porphyrin has a complex light absorption capacity, fluorescence, and other remarkable properties which are responsible for electron transfer in cytochromes, photosynthesis in chlorophyll, and oxygen binding in hemoglobin [21]. However, the low solubility of porphyrin in water always limits its clinical application. Therefore, the transformation of porphyrins into carbon dots is expected to enhance their solubility in water [22, 23].

Carbon dots (C-dots or CDs) are carbon-based nanoparticles that are less than 10 nm in size. Compared to other nanomaterials, C-dots have some unique properties, such as high solubility, fluorescence, photoluminescence, photostability, low toxicity, good biocompatibility, and the ability to present multiple functional groups on their surface. No wonder they have wide-range applications in various fields [24–26]. Carbon dots are promising to be developed as biosensors, diagnostics, and therapeutic agents for cancer and infectious diseases. The latest findings that C-dots could also be made from non-toxic natural compounds and biocompatible materials highlighted their biological application potential in the near future [25, 26]. For instance, recent studies have reported that carbon dots derived from several bioactive compounds, including curcumin, glycyrrhizic acid, and garlic (*Allium sativum*), have been shown to have antiviral effects against SARS-CoV-2 [27–29].

In the current study, we explored the antiviral potential of porphyrin and porphyrin-derived C-dots (por-CDs) against SARS-CoV-2. We assessed their antiviral activity using the DBSS for the preliminary assay and further, on Vero E6 cells as the model of mammalian cells for the in vitro evaluations. The transformation of porphyrin into carbon dots, in fact, decreased its cytotoxicity and increased its performance in disrupting SARS-CoV-2 infection in vitro by inhibiting the dimerization of the SARS-CoV-2 nucleocapsid C-terminal domain (N-CTD). Therefore, this study demonstrated the potential of porphyrin-derived C-dots as a promising COVID-19 antiviral drug. Our study is also the first to apply the DBSS for the preliminary screening of SARS-CoV-2 antiviral drugs, where we minimized the use of BSL-3 laboratory for the screening process.

## Results

### Viral protein inhibition of porphyrin through in silico analysis

The interaction of porphyrin with the SARS-CoV-2 N-CTD was analyzed in silico. The results showed that porphyrin was able to spontaneously interact with the target protein, indicated by a negative binding free energy (− 8.6 kcal/mol) of their interaction. It was also clear that the binding site of porphyrin was laid on a socket of the N-CTD which is its dimerization region (Fig. 2b). Among all residues in the region, some of them, called dimerization residues, were essential for the homodimer formation of the SARS-CoV-2 nucleocapsid, including Gln^260^, Arg^262^, Thr^263^, Ala^264^, Arg^277^, Gly^284^, and Phe^286^ [30]. From the in silico analysis, we found that 14.28% of the total dimerization residues contacted porphyrin. In particular, their interactions were dominated by hydrophobic and non-covalent interactions: van der Waals (Gln^260^, Arg^262^, Thr^263^, Arg^277^, Gly^284^, Phe^286^, Gly^287^, Gly^295^, and Thr^296^) and hydrogen bond (Ala^264^) (Fig. 2c). Leu^291^ formed an alkyl-alkyl interaction either with the pyrrole rings or the center of the porphyrin ring. Meanwhile, some residues interacted with the pyrrole rings of porphyrin through π interactions with side-by-side geometries: π-Sigma (Val^270^) and π-π stacked (Phe^274^ and Trp^301^).

### Viral protein inhibition of porphyrin and porphyrin-derived C-dots through dimer-based screening system

**Fig. 3:**
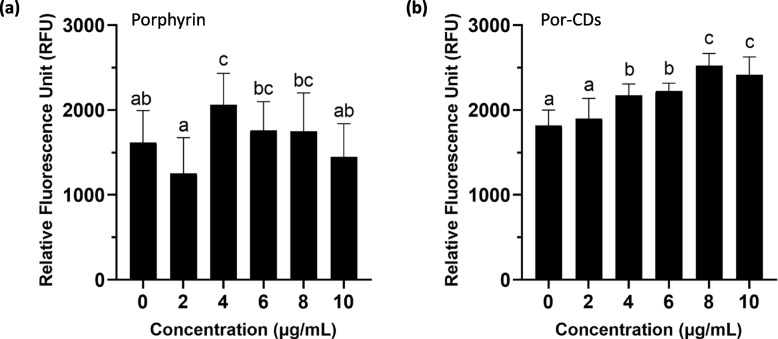
Inhibition of SARS-CoV-2 nucleocapsid dimerization represented by relative fluorescence intensity unit (RFU) emitted by genetically engineered *E. coli* after addition of porphyrin (**a**) and porphyrin-derived C-dots (por-CDs) (**b**) at varying concentrations. Relative fluorescence intensity is defined as the fluorescence intensity relative to the cell culture biomass. Data were presented as mean ± standard deviation. The significant difference between groups was tested using one-way ANOVA, followed by Tukey’s post hoc test; different letters indicate significant differences between treatments (*p* ≤ 0.05)

The antiviral activities of porphyrin and por-CDs were assessed using the dimer-based screening system (DBSS). The fluorescence signal shows the ability of tested compounds in inhibiting the dimerization of the SARS-CoV-2 N-CTD; nevertheless, the relative fluorescence values do not correspond to the amount of target protein that is inhibited. The results showed that porphyrin was able to disrupt the dimerization of the target protein at a concentration of 4–8 μg/mL, indicated by the significant differences in emitted fluorescence compared to control groups (*p* ≤ 0.05) (Fig. 3a). Yet, it was difficult to obtain consistent results between experiment replicates. We hypothesized that the low water-solubility of porphyrin affected its delivery into cells, as also suggested by Dai et al. [31]. At the same time, the addition of 4–10 μg/mL por-CDs significantly amplified the fluorescence signal compared to control groups (*p* ≤ 0.05) (Fig. 3b). It showed that por-CDs were capable of inhibiting the dimerization of N-CTD in a broader concentration range. It is worth mentioning that the same experiment for por-CDs gave more consistent results between replicates which indicated the enhancement of porphyrin solubility in water due to its structural modification into nanodot particles, which implied its bioavailability.

### Cytotoxicity and antiviral activity of porphyrin and porphyrin-derived C-dots on Vero E6 cells

The antiviral performances of the tested compounds were further proved in vitro. We assessed their cytotoxicity on African green monkey kidney cells, Vero E6, using MTT (3-[4,5-dimethylthiazol-2-yl]-2,5 diphenyl tetrazolium bromide) assay, followed by their antiviral activity on the SARS-CoV-2-infected cells based on the cytopathic effect reduction (CPER) assay. Screening hits were identified as compounds with the lowest cytotoxicity and the highest antiviral activity.

In this study, porphyrin, which is a non-polar compound, was dissolved in DMSO, a non-polar solvent known to be moderately toxic for cells. To avoid biases from the effect of DMSO on cell viability, we carefully assessed its toxicity at the concentration used in the assay. It was proven that DMSO at the concentration of 1% (*v/v*) did not affect Vero E6 cell viability compared to the untreated cells (*p* > 0.05). Otherwise, there was a negligible increase in the mean percentage of cell viability treated with 1% DMSO compared to the untreated cells. A similar phenomenon was also observed by Li et al. [32], where exposure to 1% DMSO for 72 h did not decrease cell viability; instead, it slightly promoted cell growth.

**Fig. 4:**
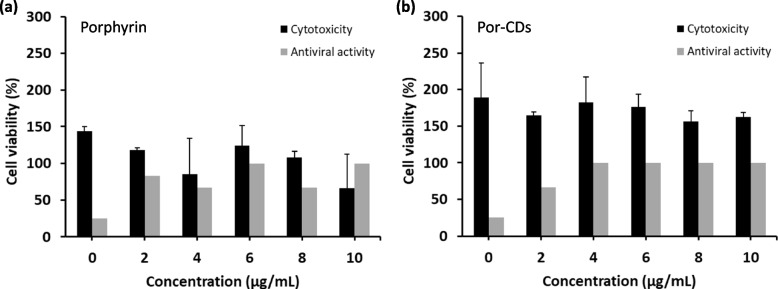
Dose-dependent cytotoxicity and antiviral activities against SARS-CoV-2 of porphyrin (**a**) and por-CDs (**b**) on Vero E6 cells. The tested compounds were exposed to the Vero E6 cells for 72 h in the conditions of (i) without virus infection to measure cytotoxicity using MTT assay (gray) and (ii) after virus infection (2000 PFU; MOI 0.1) to measure antiviral activity based on CPE reduction assay (black). For cytotoxicity, 100% means 100% of cells are viable compared to the untreated cells, while for antiviral activity, 100% means 100% of wells with no CPE observed (the calculation is shown in Table S1). Data were presented as mean ± standard deviation

After 72-h exposure, the different concentrations of porphyrin and por-CDs showed different effects on Vero E6 cells (Fig. 4). Generally, the cell viability decreased as the concentration of the tested compounds increased. As expected before, the por-CDs have lower cytotoxicity than their basic form, porphyrin. The cell viability was significantly decreased with the addition of 8 and 10 μg/mL porphyrin (*p* ≤ 0.05). In contrast, the exposure of por-CDs at the concentration of 2–10 μg/mL did not reduce the viability of Vero E6 cells significantly (*p* > 0.05). Particularly, the cell viability reduction due to the exposure of por-CDs up to 10 μg/mL for 72 h was only 26.88%, while EN-ISO 10993–5 (2009) [33] sets a 30% reduction of cell viability as a threshold for a drug candidate considered cytotoxic.

To strengthen our analysis, we calculated the 50% cytotoxicity concentration (CC_50_) of porphyrin and por-CDs on Vero E6 cells. The CC_50_ represents the concentration of drugs or substances that reduce cell viability by 50% after a certain period of exposure. The higher CC_50_ means the less toxic the substance is to cells [34]. In this study, por-CDs possessed a relatively higher CC_50_ than porphyrin, which was 55.7 μg/mL versus 15.5 μg/mL. According to the US National Cancer Institute (NCI) [35], plant crude extracts with CC_50_ < 30 μg/mL on mammalian cells after 72-h exposure were considered cytotoxic, while Zirihi et al. [36] defined plant extracts as highly cytotoxic substances if they possessed CC_50_ < 20 μg/mL. Therefore, we could conclude that por-CDs had tolerable cytotoxicity for the clinical applications on humans and in general, mammals.

Furthermore, an increase in the concentration of the compounds was proportional to the improvement of viability of the SARS-CoV-2-infected cells, showing the inhibition of viral infection (Fig. 4). Porphyrin presented the maximum antiviral activity at the concentration of 6 and 10 μg/mL, which was 75% CPE reduction. Nonetheless, the maximum antiviral activity of por-CDs was presented at the concentration of 4–10 μg/mL which also resulted in 75% cytopathic effect (CPE) reduction. Therefore, por-CDs demonstrated better performance compared to their basic forms. Por-CDs could significantly reduce the CPEs at the lowest concentration of 4 μg/mL, while the antiviral effect of porphyrin just reached its peaks at the concentration of 6 μg/mL.

Considering their cytotoxicity and antiviral potentials, we chose the optimum concentration × which has the greatest potential as well as is non-toxic to the target cells—of each compound. The optimum concentrations of porphyrin and por-CDs were 6 and 4 μg/mL, respectively. Por-CDs were shown to have lower optimum concentration against SARS-CoV-2 compared to porphyrin, suggesting that the transformation of porphyrin into carbon dots improved its antiviral properties.

### Further evaluation of antiviral activity of porphyrin-derived C-dots

**Fig. 5:**
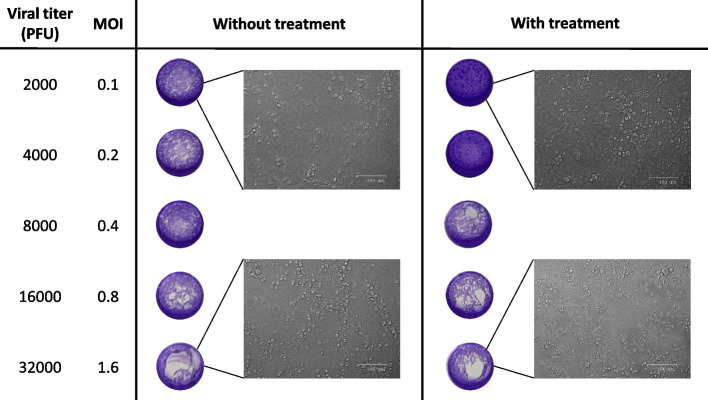
Cytopathic effect (CPE) reduction on SARS-CoV-2-infected Vero E6 cells at varying viral loads without and with 4 μg/mL porphyrin-derived C-dots (por-CDs) treatment. The appearance of CPEs in the wells after crystal violet staining is shown. The observations under inverted microscope are also shown to display CPEs on the cell monolayers. Even though the assay was performed with twelve replicates (*n* = 12), the figure presented only the representatives from each replicate. The calculation of %reduction of CPE is shown in Table 1

The 4 μg/mL por-CDs were the most promising candidates for COVID-19 antiviral drugs since they promoted CPEs reduction by 75% in the infected cells and only reduced cell viability by 6.3% in the cytotoxicity assay (Fig. 4). Therefore, we further explored the maximum viral load that could be inhibited by the compound at its optimum concentration. By increasing the viral titer, we showed a decrease in CPE reduction in the infected Vero E6 cells (Fig. 5). For Vero E6 cells infected with SARS-CoV-2 of 2000 and 4000 PFU (MOI 0.1 and 0.2, respectively), the 72-h exposure of 4 μg/mL por-CDs could reduce the cytopathic effects by more than 30% (Table 1). If the viral load was multiplied to 8000 PFU (MOI 0.4), the CPE reduction was still observed, although it was only 8.4%. Furthermore, in the case where cells were infected with 16,000 and 32,000 PFU of the virus (MOI 0.8 and 1.6, respectively), 4 μg/mL por-CDs were not effective anymore in suppressing the virus propagations as CPEs were observed in all replicates.

**Table 1:** Cytopathic effect (CPE) reduction of 4 μg/mL por-CD treatment on SARS-CoV-2-infected Vero E6 cells at varying viral loads. The %CPE was the percentage of wells with observed CPEs. The assay was performed with twelve replicates (*n* = 12)

Viral titer (PFU)	MOI^a^	%CPE		%Reduction of CPE
		**Without treatment**	**With treatment**	
2000	0.1	50	16.6	33.4
4000	0.2	66.6	33.3	33.3
8000	0.4	100	91.6	8.4
16,000	0.8	100	100	0
32,000	1.6	100	100	0

^a^*MOI* multiplicity of infection

## Discussion

This study demonstrated the potential of porphyrin, one of the abundant bioactive compounds in nature, to be developed as an antiviral drug for COVID-19. Natural products, especially herbal or plant extracts, give a promising alternative for treating various diseases. Particularly, several phytochemicals have been known for their antiviral activity, for instance, curcumin from turmeric (*Curcuma longa*), rutin from weeping fig (*Ficus benjamina*), and also epigallocatechin gallate from ginseng (*Panax ginseng*) and tea (*Camelia sinensis*) [37]. Compared to synthetic drugs, natural-based medicines have milder adverse effects and a lower risk in developing resistance for long-term uses. However, common organic compounds have poor solubility in water, limiting their delivery and bioavailability. To overcome those constraints, different drug delivery systems have been developed, such as micelles, liposomes, microspheres, and nanoparticles [37, 38]. As demonstrated by Ragab et al. [39], encapsulating chrysin, a natural flavone with anticancer activity, within a chitosan nanoparticle enhances its bioactivity in vitro. Therefore, this study also investigated the biological activity improvement of nanoparticles, specifically carbon dots, derived from porphyrin in disrupting SARS-CoV-2 infection.

Based on the in silico analysis, porphyrin displayed a potential to inhibit the dimerization of SARS-CoV-2 nucleocapsid. Porphyrin could spontaneously bind to the SARS-CoV-2 N-CTD (binding affinity energy of − 8.6 kcal/mol). The value is comparable to several COVID-19 antiviral candidates targeting the same viral protein, including silmitasertib (− 7.89 kcal/mol), fedratinib (− 8.2 kcal/mol), nintedanib (− 8.4 kcal/mol), dovitinib (− 8.6 kcal/mol), and rapamycin (− 8.9 kcal/mol), even higher than TMCB (− 7.05 kcal/mol), lopinavir (− 6.58 kcal/mol), sapanisertib (− 6.14 kcal/mol), chloroquine (− 5.62 kcal/mol), Arbidol (− 5.32 kcal/mol), oseltamivir (− 5.08 kcal/mol), ribavirin (− 4.86 kcal/mol), favipiravir (− 4.44 kcal/mol), hydroxychloroquine (− 4.32 kcal/mol), and remdesivir (− 3.46 kcal/mol) [40–42]. Further, as shown in Fig. 2c, porphyrin specifically binds to several key residues that can also interact with other COVID-19 antiviral candidates targeting the CTD of nucleocapsid [32, 33]. According to Zhou et al. [30], there were five regions on the dimerization interface of SARS-CoV-2 nucleocapsid subunits where strong hydrogen bonds were formed to maintain a stable dimer conformation. The residues bound to porphyrin were located in those regions: Gln^260^, Arg^262^, Thr^263^, and Ala^264^ in region I, while Arg^277^, Gly^284^, and Phe^286^ in region II. Notably, Arg^277^ is highly conserved in the Nucleocapsid of the coronavirus family, indicating its fundamental role in protein dimerization [30]. Thus, binding of porphyrin to those residues theoretically disrupts the functionality of Nucleocapsid as well as the viral propagation in the host cells.

Previous studies have reported different modes of actions of porphyrin derivatives and carbon dots as antiviral agents, such as inhibition of viral particle binding to its receptors, preventing the viral attachment and entry, inhibiting viral proteases, and also disruption of viral replication [43–48]. However, our DBSS results confirmed that the tested compounds could specifically target the SARS-CoV-2 nucleocapsid by inhibiting its homodimer formation (Fig. 3). Nucleocapsid is critical for coronaviruses, where it is involved in genome packaging as well as viral replication and assembly. To be functional, it must be in a homodimer state [49]. Thus, inhibition of the dimerization of nucleocapsid is a potential target for COVID-19 antiviral drugs. Nevertheless, we do not deny that the tested compounds might have more than one mechanism in disrupting SARS-CoV-2 infection in vitro. Further investigation is needed to explore the other possible targets of porphyrin and por-CDs.

It is worth mentioning that this study demonstrated the usefulness of target-based drug screening platforms, which provides us insights into the specific mechanism of antiviral drug candidates and could also be applied in studying molecular mechanisms of viral or bacterial proteins. In addition, this study also highlighted the practical uses of the DBSS as a platform for research and drug screening without involving the infectious agents directly. Therefore, it would be possible to study infectious diseases with lower biological risks, minimizing the need for high biosafety level laboratory facilities in responding to future emergency events, such as pandemics [3, 50]. In the case of the COVID-19 pandemic, this concern has led to the development of various drug screening systems without the use of live SARS-CoV-2 virus. For example, Chen et al. [51] designed a drug screening system exploring the − 1 programmed ribosomal frameshifting (PRF) of SARS-CoV-2 replication by using the lentiviral vector. Luo et al. [52] developed a non-infectious replicon system for SARS-CoV-2 that could be applied for a safe COVID-19 drug screening. Yuan et al. [53] demonstrated the ability to screen drugs inhibiting SARS-CoV-2 replication using pseudovirus, thus greatly reducing the risk of infection during experiments. For future works, we suggested the application of this coronaviral nucleocapsid-targeting DBSS approach to explore the antiviral activities of different types of bioactive compounds other than phytochemicals, such as microbial metabolites [54], and for drug repurposing endeavors [40–42].

Furthermore, this study evaluated the cytotoxicity and antiviral activity of porphyrin and por-CDs against SARS-CoV-2 in mammalian cells. On Vero E6 cells, the CC_50_ of porphyrin used in this study (15.5 μg/mL, equivalent to 49.94 μM where the molecular weight of porphyrin is 310.352 g/mol) was much higher than the other porphyrin derivatives: verteporfin (10.33 μM) [43], Sn-protoporphyrin IX (20.7 μM) [55], and a novel meso-arylporphyrin (43 μM) [56]. Nonetheless, it is still below the standards for cytotoxicity of plant extracts on mammalian cells [35, 36]. Therefore, our results strongly indicated that por-CDs had a better prospect to be developed further as COVID-19 antiviral drugs. Compared to its basic form, porphyrin-derived C-dot was less toxic to cells and exhibited better performances in suppressing SARS-CoV-2 propagation in vitro (Fig. 4). In the same manner, Lin et al. [57] demonstrated a lower cytotoxicity and a higher antiviral activity of curcumin-carbon dots compared to curcumin. Tong et al. [28] also reported that the transformation of glycyrrhizic acid into carbon dots reduced its cytotoxicity in vitro.

One of the most rational reasons for the better antiviral activity of porphyrin-derived carbon dots is due to their better interaction with the target protein. Indeed, since the molecular structure of por-CDs was not available yet, we could not perform any in silico analysis to justify this hypothesis. For our reference, Tong et al. [28] observed that glycyrrhizic acid-derived carbon dots had a larger surface area and more contact sites compared to glycyrrhizic acid, while still keeping the functional groups of glycyrrhizic acid on its surface. Therefore, we confidently predicted that por-CDs could interact better with the target viral protein and possess an enhanced antiviral activity than porphyrin itself.

Finally, we tried to explore further the performance of por-CDs as novel antiviral drug candidates for COVID-19. We determined 4 μg/mL as the optimum concentration of por-CDs which promoted CPEs reduction by 75% in the infected cells and only reduced cell viability by 6.3% in the cytotoxicity assay (Fig. 4). As shown in Table 1, treatment of 4 μg/mL por-CDs for 72 h was able to reduce CPEs in the infected cells by more than 30% with the viral titer up to 4000 PFU. This antiviral capacity is comparable to other porphyrin derivatives, protoporphyrin IX and verteporfin, reported by Gu et al. [44].

Complementing the proof-of-concept demonstrated in this study, further pharmacological evaluation is still required to gain a more comprehensive understanding of the mechanism of por-CDs in disrupting SARS-CoV-2 infection. Exploration of other mechanisms of action of the antiviral drug candidate is imperative, as shown by a similar experiment by Marín-Palma et al. [58]. Furthermore, in vivo assessment is also mandatory, as they may result in different levels of toxicity and antiviral activity [59].

To the best of our knowledge, our study is the first to utilize carbon dots to increase the performance of porphyrin as a SARS-CoV-2 antiviral drug. However, the other applications of por-CDs could be explored further. It has been known that carbon dots possessed a fluorescence activity, thus making them capable of being used in many biomedical applications, including diagnostics, biosensors, photoacoustic imaging, therapeutics, and the simultaneous therapy/imaging applications called theranostics [25, 26, 60–62]. As shown in the previous studies, due to their great penetration capacity into cells, carbon dots increased the antimicrobial efficiency of porphyrin [63] and also improved its anticancer activity in photodynamic and photothermal therapy [64, 65]. Following a similar approach, future prospective studies would be explorations of various natural compound-derived carbon-dots that have the potential to be developed as antiviral, antibacterial, or even anticancer drugs.

## Conclusions

This study discovered the potential of porphyrin-derived C-dots to be developed as safe and effective antiviral drugs for COVID-19. Using the DBSS targeting the CTD of SARS-CoV-2 nucleocapsid, porphyrin demonstrated a dimerization inhibitory activity against the N-CTD at a concentration range of 4–8 μg/mL; however, por-CDs were able to prevent the viral antigen dimerization in a broader concentration range, which was 4–10 μg/mL. The improved performances of por-CDs were further assessed in Vero E6 cells, where the por-CDs were shown to possess a lower cytotoxicity than porphyrin. Exposuring por-CDs at a concentration range of 2–10 μg/mL for 72 h only reduced the cell viability by as much as 26.88%, which surpassed the international standards for drug cytotoxicity, indicating that the antiviral drug candidate was non-toxic. For comparison, a significant viability reduction was observed in the Vero E6 cells exposed to porphyrin above the concentration of 6 μg/mL. Por-CDs also demonstrated better performance in disrupting SARS-CoV-2 infection in vitro compared to porphyrin. At the lowest concentration of 4 μg/mL, por-CDs significantly suppressed CPEs (75% reduction) in infected cells. Meanwhile, in the cells treated with porphyrin, a similar output was generated at the concentration of 6 μg/mL, suggesting an improvement of antiviral capacity after transforming porphyrin into carbon dots particles. Nevertheless, since it was still a proof-of-concept experiment, further assessments are required to ensure their safety and effectiveness in treating COVID-19 in humans. Overall, this study demonstrated the biological activity improvements of bioactive compounds in the form of C-dots. In addition, this study validated DBSS as a reliable novel drug screening platform that can be used under limited access to high -containment laboratory facilities, so that the similar approach can be applied to other natural compounds and promising drug candidates to treat other diseases.

## Materials and methods

### Cells and virus

Genetically engineered *Escherichia coli* BL21 (DE3) expressing a modified SARS-CoV-2 nucleocapsid as the dimer-based screening system (DBSS) was established by Fibriani et al. [18]. The bacteria culture was maintained in Luria–Bertani (LB) agar medium containing 200 μg/mL ampicillin and refreshed every 2 weeks. The bacterial cell stock was preserved at − 80 °C. Maintenance and experiments involving the engineered *E. coli* cells were conducted at School of Life Sciences and Technology, Institut Teknologi Bandung, Indonesia.

Vero E6 cell line was maintained in Dulbecco’s modified eagle medium (DMEM, Sigma-Aldrich, USA) supplemented with 10% heat-inactivated fetal bovine serum (FBS, Gibco, USA) and 1% penicillin/streptomycin (Sigma-Aldrich, USA). Every 2–4 days, the confluent cell monolayers were harvested by trypsinization and seeded into a new vessel. The cells were used for cytotoxicity and antiviral activity assays once they reached a passage number of 3.

SARS-CoV-2 (GISAID Accession ID: EPI_ISL_4004658) was isolated from Bogor, Indonesia, in May 2020. The virus was propagated in the Vero E6 cell line and the viral titer was determined using 50% tissue culture infectious dose (TCID50) assay. The TCID50/mL value was converted into plaque-forming unit (PFU)/mL by dividing it by 0.7 [66]. The viral culture was preserved at − 80 °C. All experiments involving viruses were conducted in a certified BSL-3 biocontainment facility at the Indonesian National Research and Innovation Agency (BRIN), Indonesia, and the experiment protocols have been approved by the Biosafety and Ethics Committees of BRIN.

### Compound preparation

Porphyrin (C_20_H_14_N_4_; CAS No. 101–60-0) was purchased from Muse Chemicals (Cat No. M071749, USA) in the form of powder. Porphyrin was solubilized in 100% dimethyl sulfoxide (DMSO) and preserved at − 20 °C, protected from light. Meanwhile, porphyrin-derived C-dots (por-CDs) were prepared through a solvothermal method as follows. Precursor solution was made by stirring 1 M citric acid (C_6_H_8_O_7_; CAS No. 77–92-9) and 5 M urea (CH_4_N_2_O; CAS No. 57–13-6) in distilled water for 15 min at room temperature. Separately, 0.03 M porphyrin was diluted in DMSO. Then, the porphyrin and precursor solution were transferred into the Teflon inner autoclave and heated for 5 h at 160 °C. The solution was centrifuged, filtered by RC filter 0.22 μm, and freeze-dried for 3 days. In preparation for assays, the freeze-dried por-CDs were solubilized in sterile double-distilled water. The freeze-dried por-CDs were preserved at room temperature and protected from light, while the por-CDs solution was preserved at − 20 °C.

For the viral protein inhibition assay (DBSS), all tested compounds were prepared in their appropriate solvents to stock concentrations of 100 and 500 μg/mL. For the cytotoxicity assay, all tested compounds were diluted in complete DMEM supplemented with 10% FBS to final concentrations of 2, 4, 6, 8, and 10 μg/mL. Meanwhile, for the antiviral activity assay, complete DMEM supplemented with 2% FBS was used rather than 10%.

In the DBSS procedure, the final concentration of DMSO was 5% (*v/v*). For up to 6 h, exposure to 5% DMSO only gave moderate effects on the growth of *E. coli* cells [67]. On the other hand, since DMSO concentration of more than 1% is considered toxic for animal cells [68], the final concentration of DMSO for all assays using Vero E6 cells was 1% (*v/v*).

### In silico analysis

#### Protein and ligand

The crystal structure of the C-terminal domain (CTD) of SARS-CoV-2 nucleocapsid phosphoprotein (PDB ID: 6ZCO) was retrieved from the Protein Database (https://www.rcsb.org/), deposited by Zinzula et al. [69]. The molecular structure of porphyrin (PubChem ID: 66868) was retrieved from PubChem (https://pubchem.ncbi.nlm.nih.gov/). All files were formatted to.pdbqt for molecular docking using AutoDock Tools 1.5.6 [70].

#### Molecular docking

The interaction of the tested compounds and the target viral protein was modeled using a molecular docking approach. Porphyrin was docked to the SARS-CoV-2 N-CTD using AutoDock Vina [71]. Grid box was constructed with dimensions of 36 × 38 × 34 Å and coordinates [2.482 Å, − 5.594 Å, − 1.035 Å] in a spacing of 1.00 Å. The percentage of interaction (%interaction), which is the percentage of dimerization residues of CTD of SARS-CoV-2 nucleocapsid that interacted with the ligand, was calculated as follows.

$$\mathrm{\%Interaction }= \frac{\mathrm{Number\;of\;residues\;of\;the\;protein\;interacted\;with\;the\;ligands }}{\mathrm{Total\;number\;of\;residues\;of\;the\;protein}} \times 100\%$$

In addition, the docking results were visualized using BIOVIA Discovery Studio (BIOVIA, USA).

### Viral protein inhibition assay using dimer-based screening system

The viral protein inhibition assay using the dimer-based screening system (DBSS) was described by Fibriani et al. [18]. Briefly, the assay for porphyrin and por-CDs was performed as follows. A single colony of the engineered *E. coli* BL21 (DE3) was inoculated into 5-mL Luria–Bertani (LB) broth containing 200 μg/mL ampicillin and activated by 8-h incubation at 37 °C with 150 rpm shaking, followed by overnight incubation. The activated bacterial culture was transferred to fresh LB broth with an inoculum of 10% (*v/v*) and incubated at 37 °C with shaking until it reached optical densities (OD_600_) of 0.4. After the addition of 0.4 μM IPTG, the bacterial culture was transferred to 96-well plates (95 μL/well). Then, the tested compounds were added (5 μL/well), followed by 4-h incubation at 37 °C with shaking. As vehicle control (0 μg/mL porphyrin treatment), cells were treated with 5% DMSO. After the incubation, the cell culture optical density was read at 600 nm using GloMaxⓇ Explorer (Promega, USA), followed by fluorescence intensity measurement (excitation at 475 nm and emission at 500–550 nm). Relative fluorescence intensity is defined as the fluorescence intensity relative to the culture biomass at OD600. The optical density and fluorescence intensity values of each sample were normalized by blank, then the relative fluorescence intensity was calculated as follows.

$$\mathrm{Relative\;fluorescence\;intensity }= \frac{\mathrm{normalized\;fluorescence\;intensity}}{\mathrm{normalized\;cell\;culture\;optical\;density}}$$

### Cytotoxicity assay on Vero E6 cells

The cytotoxicity of porphyrin and por-CDs on Vero E6 cells was evaluated using the MTT (3-[4,5-dimethylthiazol-2-yl]-2,5 diphenyl tetrazolium bromide) assay, which quantifies the viable cells after treatment of the tested compounds. Vero E6 cells were seeded in 96-well plates (100 μL/well) at a density of 2 × 10^4^ cells/well and incubated at 37 °C with 5% CO_2_. After incubation for 24 h, the culture medium was discarded, followed by the addition of the diluted compounds to cell monolayers (100 μL/well). For 0 μg/mL porphyrin treatment (vehicle control), cells were treated with 1% DMSO-contained medium. The assay was performed with three replicates (*n* = 3). The plates were incubated for 72 h at 37 °C with 5% CO_2_. At the end of the treatment, the supernatant was discarded, then the cells were washed with phosphate buffer saline (PBS). MTT (0.5 mg/mL) was added 100 μL/well and incubated for 3 h, at 37 °C with 5% CO_2_, in dark condition. Finally, 100% DMSO was added (100 μL/well) to dissolve the formazan crystals formed. After 10-min incubation at room temperature, the absorbance was read at 570 nm using Varioskan™ LUX Multimode Microplate Spectrophotometer (ThermoScientific, USA). The absorbance value of each sample was normalized by blank before the cell viability was calculated as follows.

$$\mathrm{Cell\;viability }(\mathrm{\%}) = \frac{\mathrm{normalized\;absorbance\;of\;samples}}{\mathrm{normalized\;absorbance\;of\;untreated\;cells}} \times 100\mathrm{\%}$$

The tested compounds at certain concentrations which resulted in cell viability of ≥ 70% were considered non-toxic [33]. The 50% cytotoxicity concentration (CC_50_) values of porphyrin and por-CDs were obtained by linear regression calculation.

### Antiviral activity assay against SARS-CoV-2 on Vero E6 cells

In order to examine the antiviral activity of porphyrin and por-CDs in vitro against SARS-CoV-2, we challenged the virus-infected Vero E6 cells with the tested compounds. The antiviral activity was determined based on the reduction of the cytopathic effects (CPEs) on the treated infected cells, compared with the untreated infected cells [72, 73].

The confluent Vero E6 cell monolayers (2 × 10^4^ cells/well) were then infected with SARS-CoV-2 at MOI (multiplicity of infection) of 0.1, equal to 2000 PFU/well, in 100 μL DMEM supplemented with 2% FBS and incubated for 1 h at 37 °C. To allow maximum viral adsorption, the plate was shaken every 15 min. After the incubation, the inoculum was removed. The diluted compounds were added to the infected cells (100 μL/well), followed by incubation for 72 h at 37 °C with 5% CO_2_. The untreated (0 μg/mL compounds) non-infected cells were used as the cell viability control (minimal CPEs), while the untreated infected cells were used as the cell infection control (maximum CPEs). For the vehicle control of porphyrin treatment, 1% DMSO-contained medium was used. The assay was performed with six replicates (*n* = 6). Meanwhile, the cells were fixed with 4% formaldehyde and stained with 0.5% crystal violet (CV) solution. CPE on each well was observed under inverted microscope before and after CV staining.

Furthermore, we explored the maximum viral titer that could be inhibited by the tested compounds at the optimum non-toxic concentrations. The assay was performed as the previous step, except the Vero E6 cells were infected with varying titers of SARS-CoV-2: 32,000, 16,000, 8000, 4000, and 2000 PFU/well, corresponding to MOI of 1.6, 0.8, 0.4, 0.2, and 0.1. The assay was performed with twelve replicates (*n* = 12).

### Data and statistical analysis

All data processing and statistical analysis were performed using Microsoft Excel (Microsoft, USA) and SPSS Statistics (IBM, USA). Data were presented as mean ± standard deviation. Statistical differences were tested using Student’s *t*-test for parametric data or chi-square test for non-parametric data, where *p* ≤ 0.05 was considered significantly different.

For the DBSS, the normalized fluorescence values for control and treatment groups were analyzed for homogeneity using Levene’s test. Data that had been confirmed as homogeneous were analyzed for significance using one-way analysis of variance (ANOVA), followed by post hoc analysis using Tukey’s test, where *p* ≤ 0.05 was considered significantly different.

### Supplementary Information

**Additional file 1: Table S1.** Calculation of dose-dependent antiviral activities against SARS-CoV-2 of porphyrin and por-CDs on Vero E6 cells based on cytopathic effect (CPE) observation. The tested compounds were exposed to the Vero E6 cells for 72 h after virus infection (2000 PFU; MOI 0.1).

## Data Availability

The crystal structure of the C-terminal domain (CTD) of SARS-CoV-2 nucleocapsid phosphoprotein (PDB ID: 6ZCO) is available in Protein Database (https://www.rcsb.org/). The structure of porphyrin (PubChem ID: 66,868) is available in PubChem (https://pubchem.ncbi.nlm.nih.gov/).

## Abbreviations

BSL: Biosafety level
CC_50_: 50% Cytotoxicity concentration
CDs: Carbon dots
COVID-19: Coronavirus disease-2019
CPE: Cytopathic effect
DBSS: Dimer-based screening system
MOI: Multiplicity of infection
N-CTD: Nucleocapsid C-terminal domain
PFU: Plaque forming unit
Por-CDs: Porphyrin-derived carbon dots
RFU: Relative fluorescence unit
SARS-CoV-2: Severe acute respiratory syndrome-coronavirus-2
TCID50: 50% Tissue culture infectious dose
