# Supplementary material for: Porphyrin-derived carbon dots for an enhanced antiviral activity targeting the CTD of SARS-CoV-2 nucleocapsid
Source: J Genet Eng Biotechnol. 2023 Oct 6;21:93. doi: 10.1186/s43141-023-00548-z (PMC10558421; doi:10.1186/s43141-023-00548-z)
Supplement: Supplementary file 1 — Additional file 1: Table S1. Calculation of dose-dependent antiviral activities against SARS-CoV-2 of porphyrin and por-CDs on Vero E6 cells based on cytopathic effect (CPE) observation. The tested compounds were exposed to the Vero E6 cells for 72 h after virus infection (2000 PFU; MOI 0.1). [file 43141_2023_548_MOESM1_ESM.docx]

**Table S1.** Calculation of dose-dependent antiviral activities against SARS-CoV-2 of porphyrin and por-CDs on Vero E6 cells based on cytopathic effect (CPE) observation. The tested compounds were exposed to the Vero E6 cells for 72 h after virus infection (2000 PFU; MOI 0.1).

| **Compounds** | **Concentration (µg/mL)** | **CPE Observation** | **No of wells with no CPE observed** | **Cell viability***  **(%)** |
| --- | --- | --- | --- | --- |
|  |  |  |  |  |
| Porphyrin | 0 (control) | 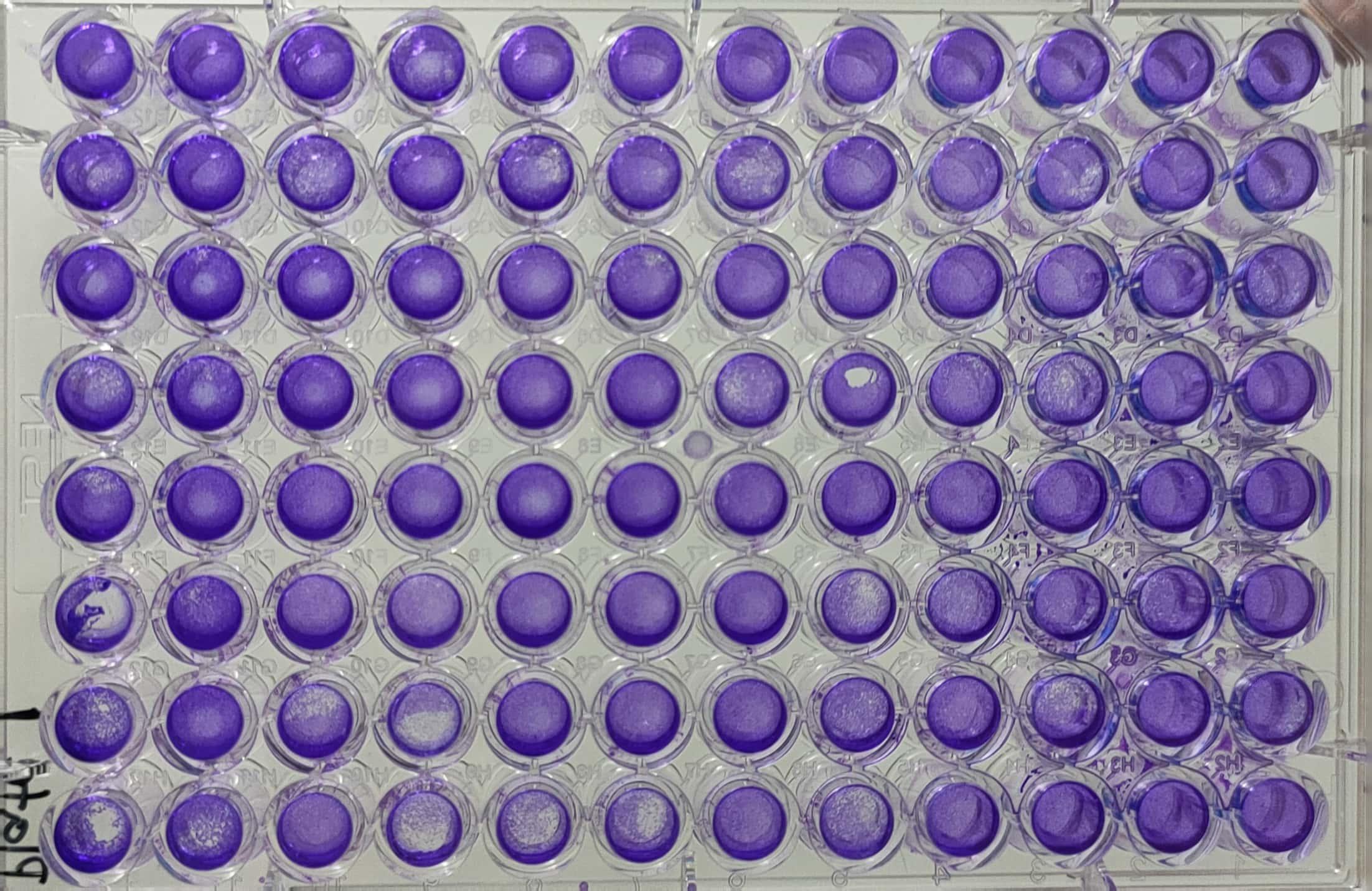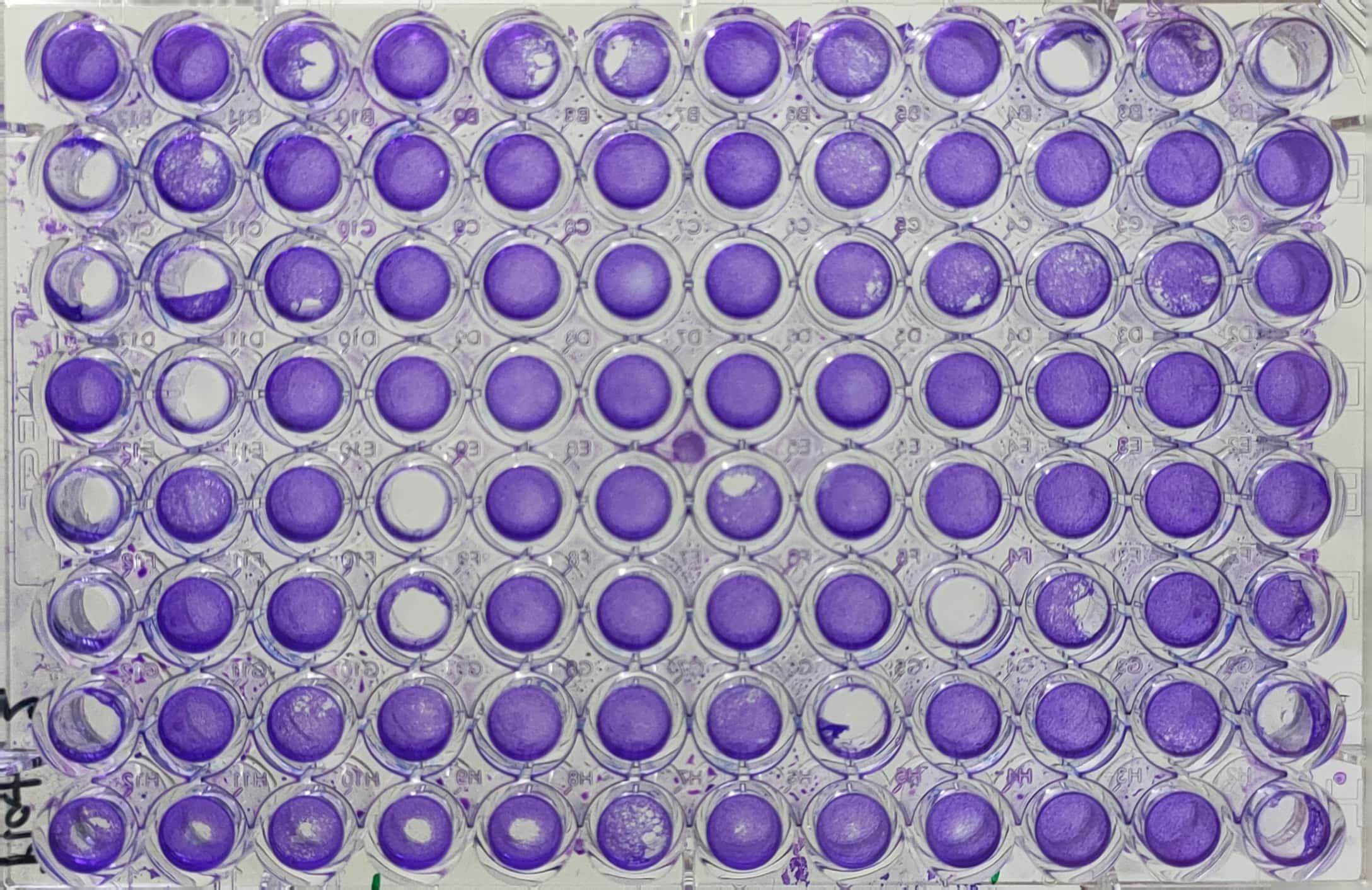 | 3/12 | 25 |
|  | 2 | 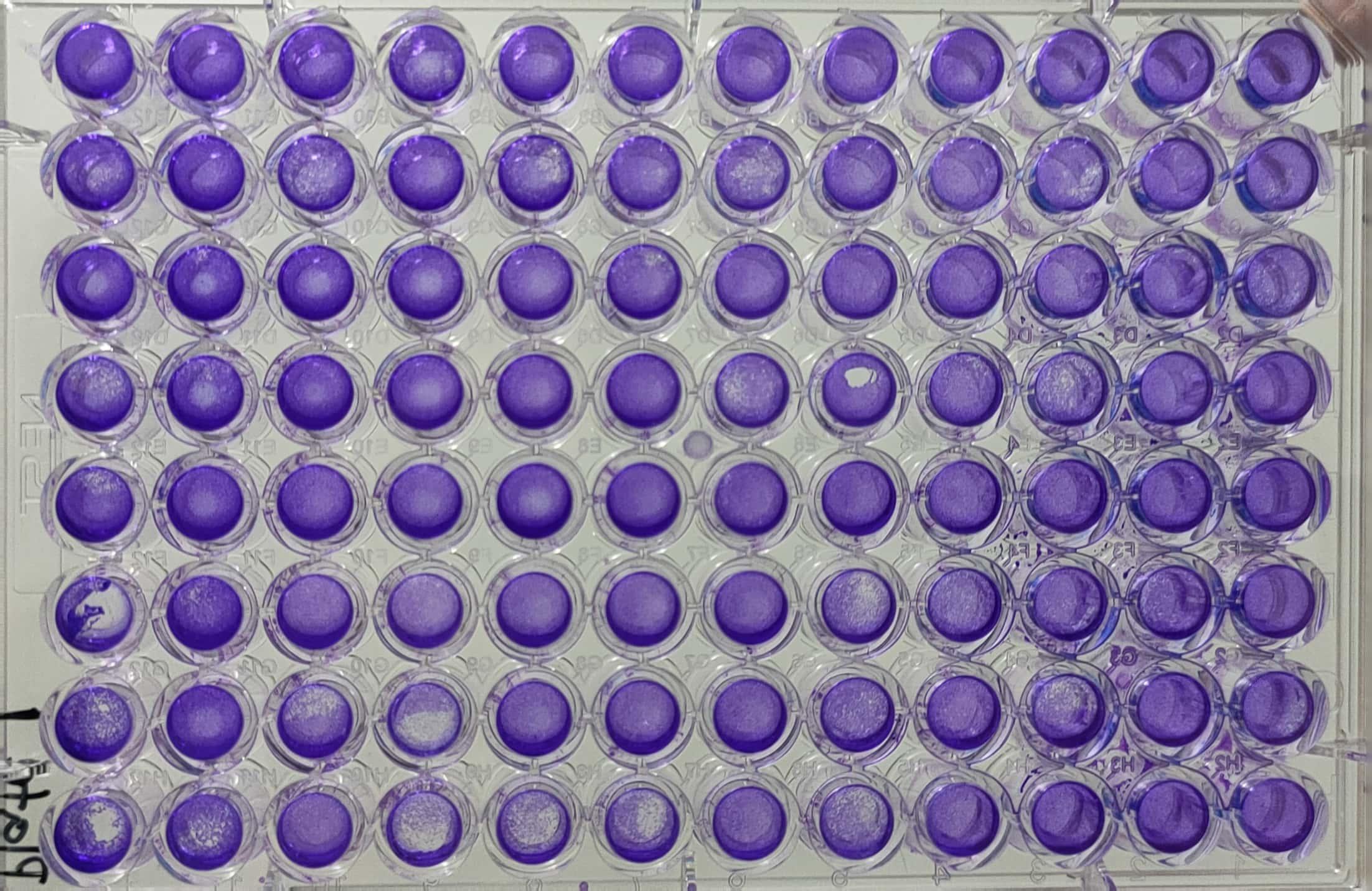 | 5/6 | 83.3 |
|  | 4 | 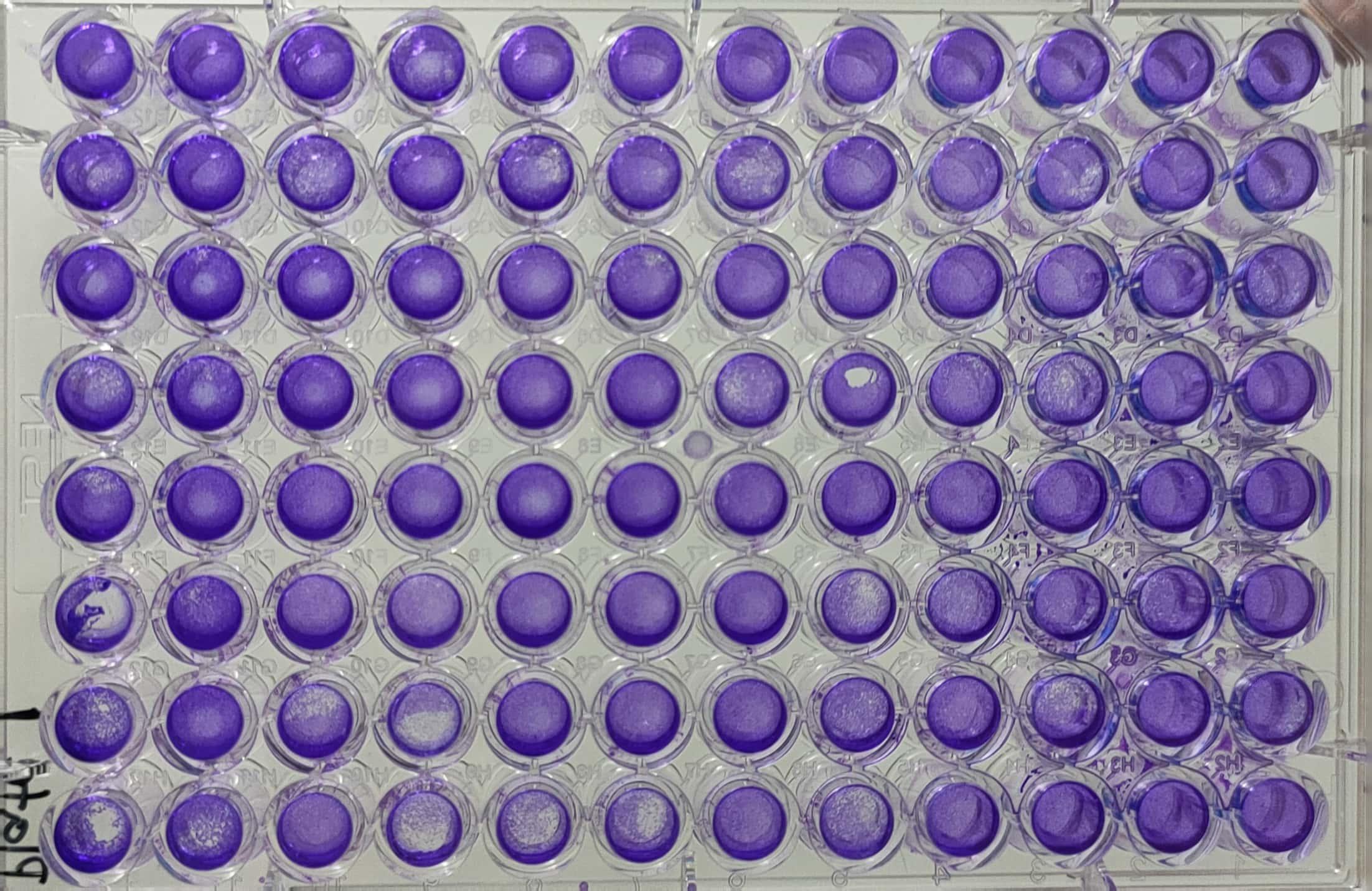 | 4/6 | 66.7 |
|  | 6 | 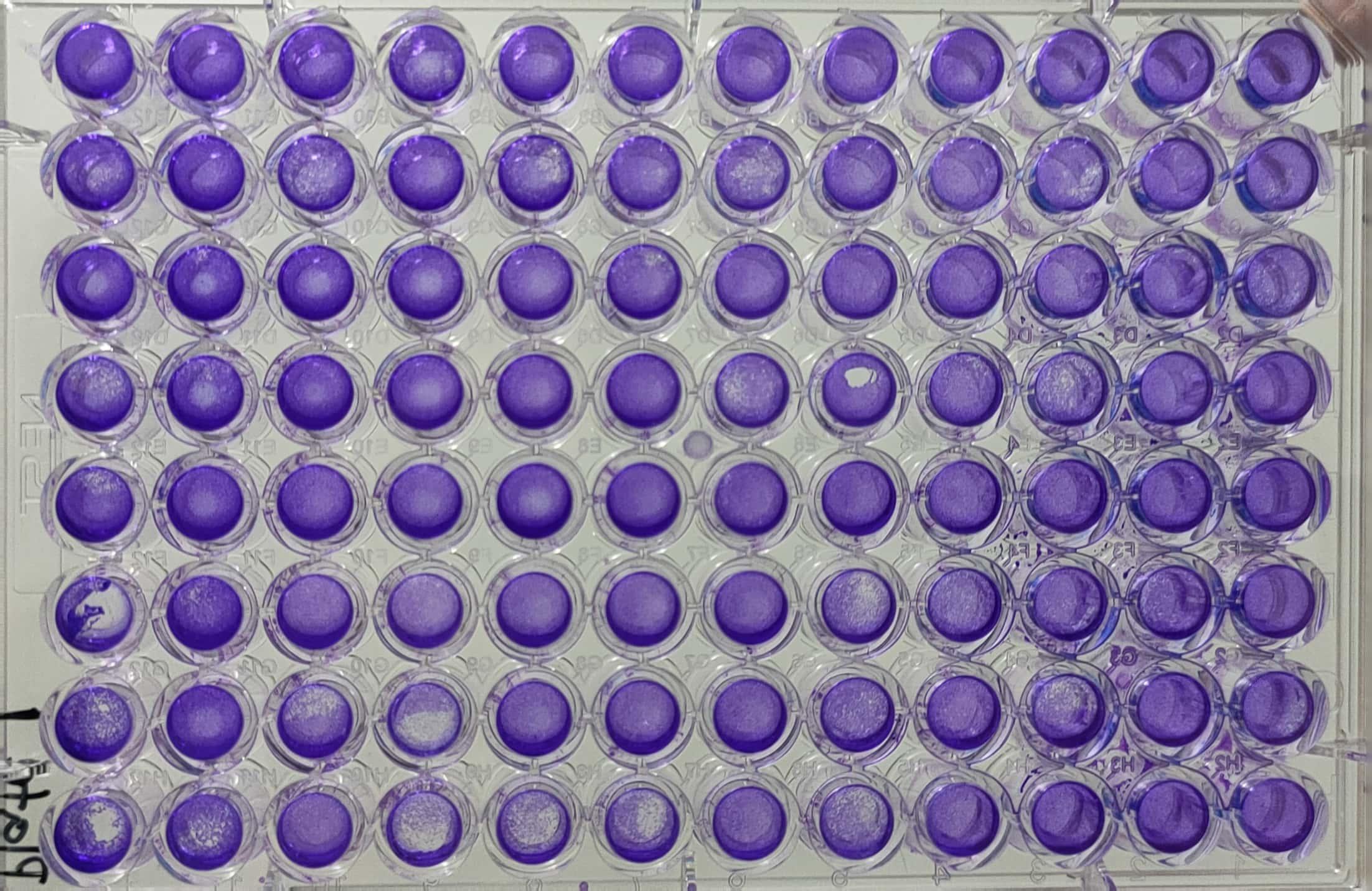 | 6/6 | 100 |
|  | 8 | 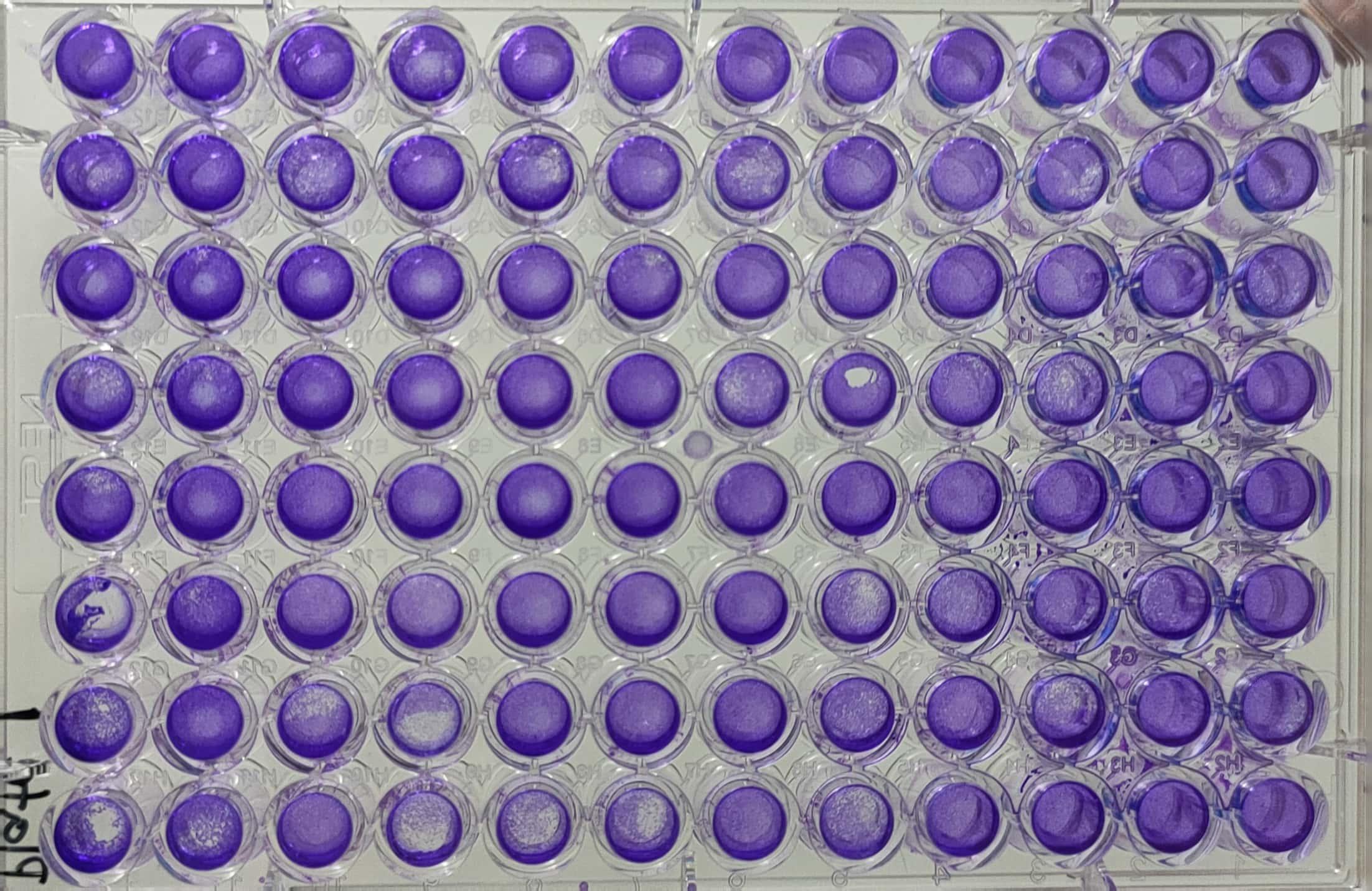 | 4/6 | 66.7 |
|  | 10 | 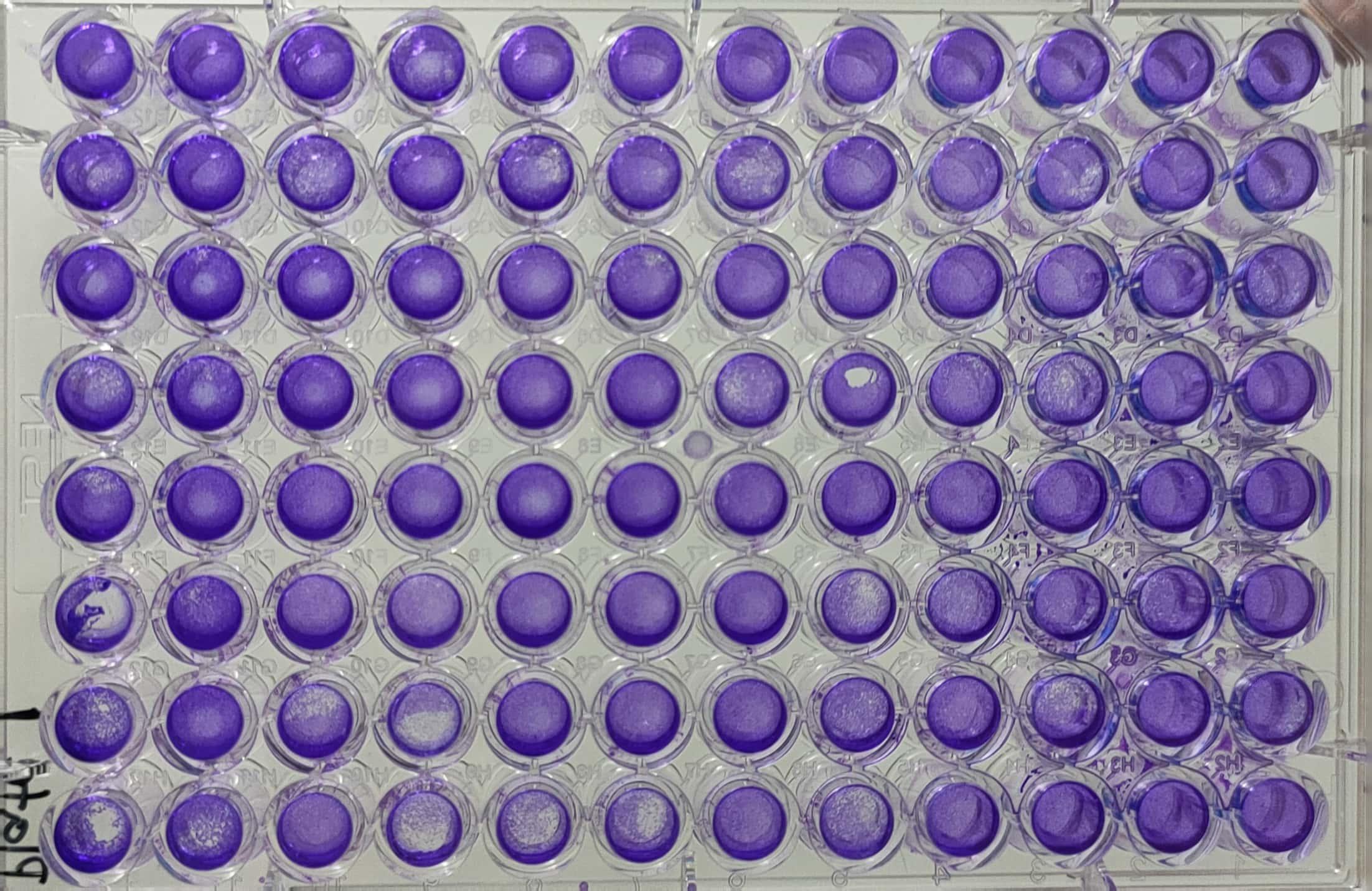 | 6/6 | 100 |
| Por-CDs | 0 (control) | 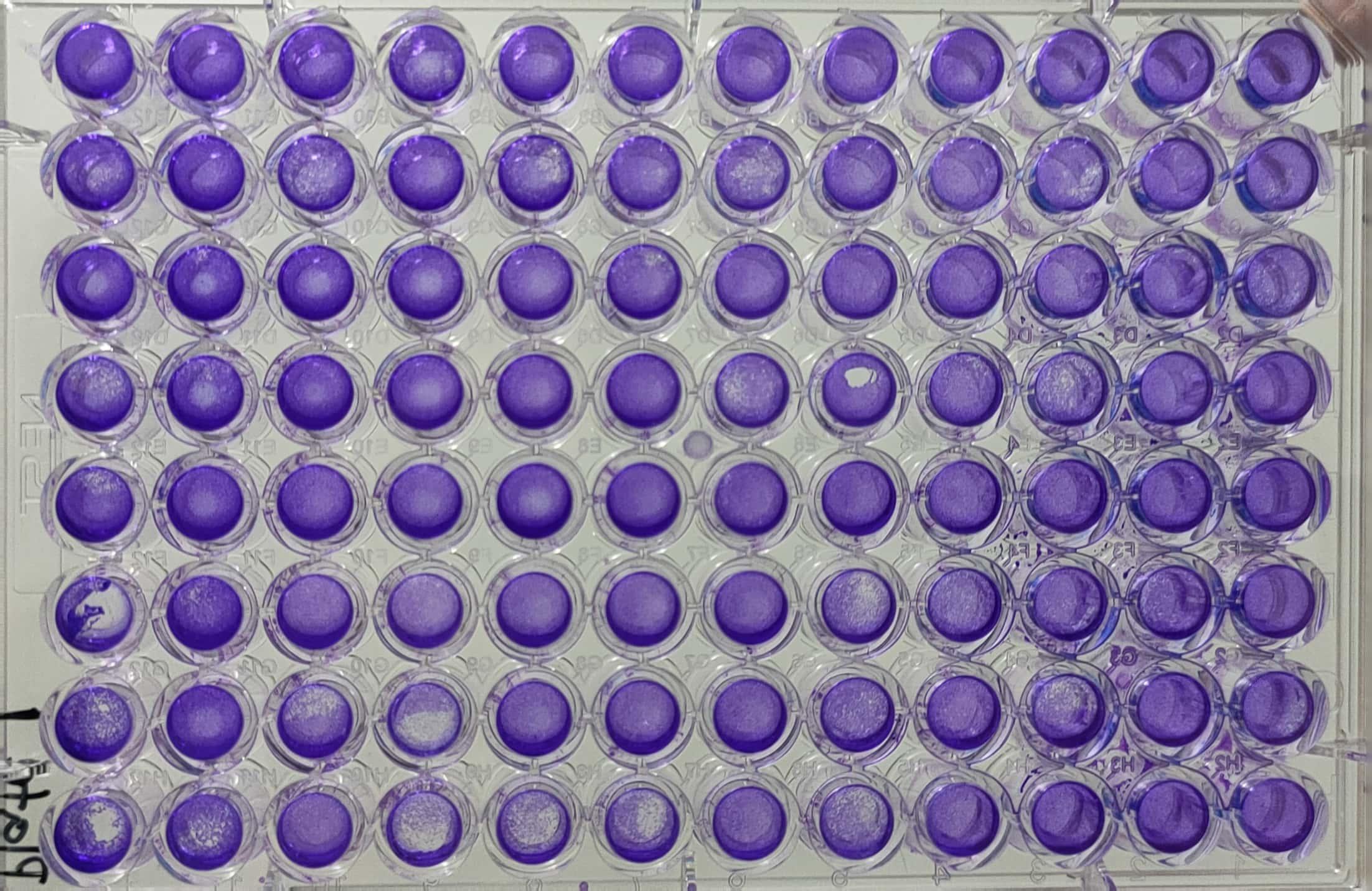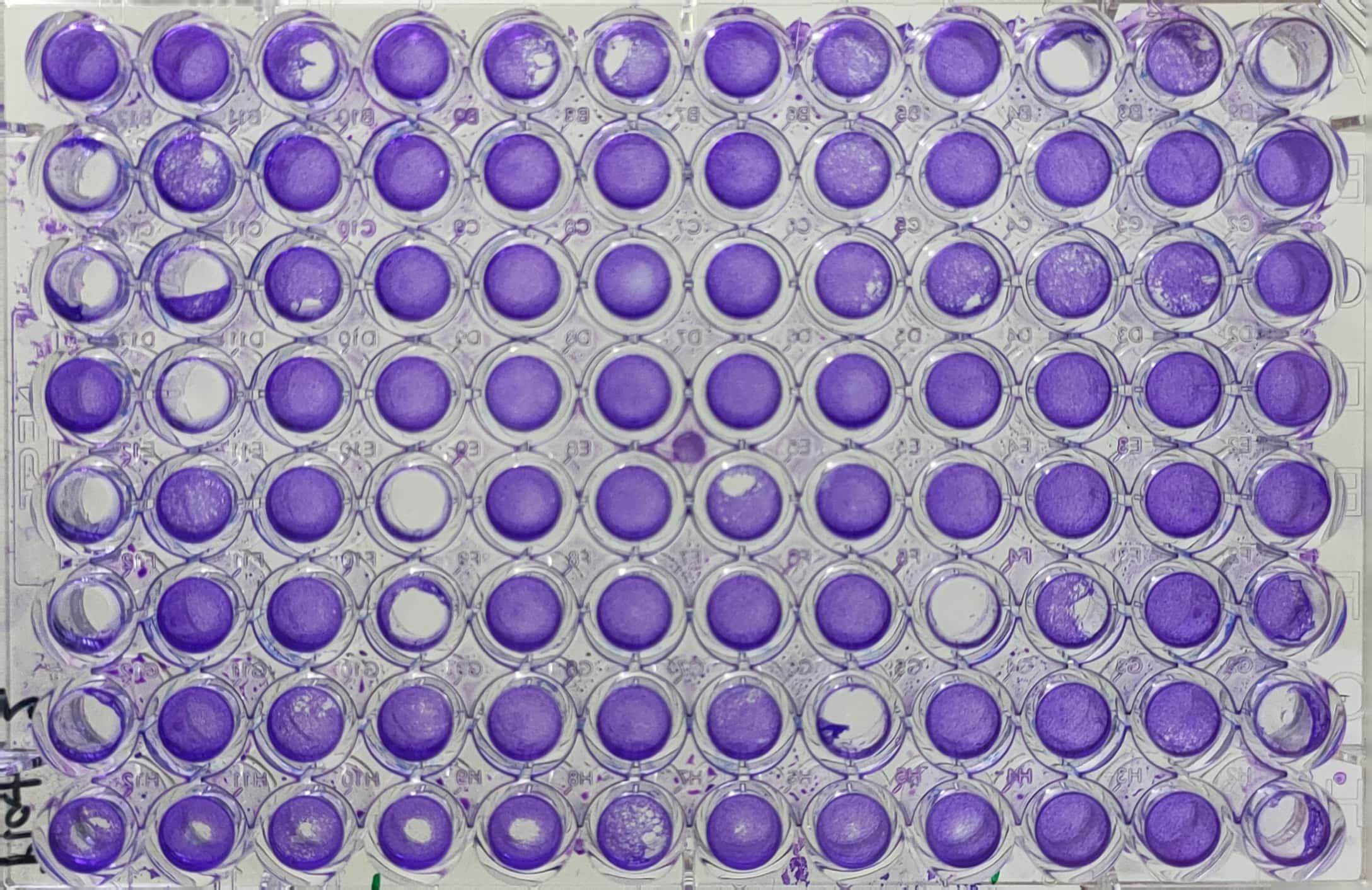 | 3/12 | 25 |
|  | 2 | 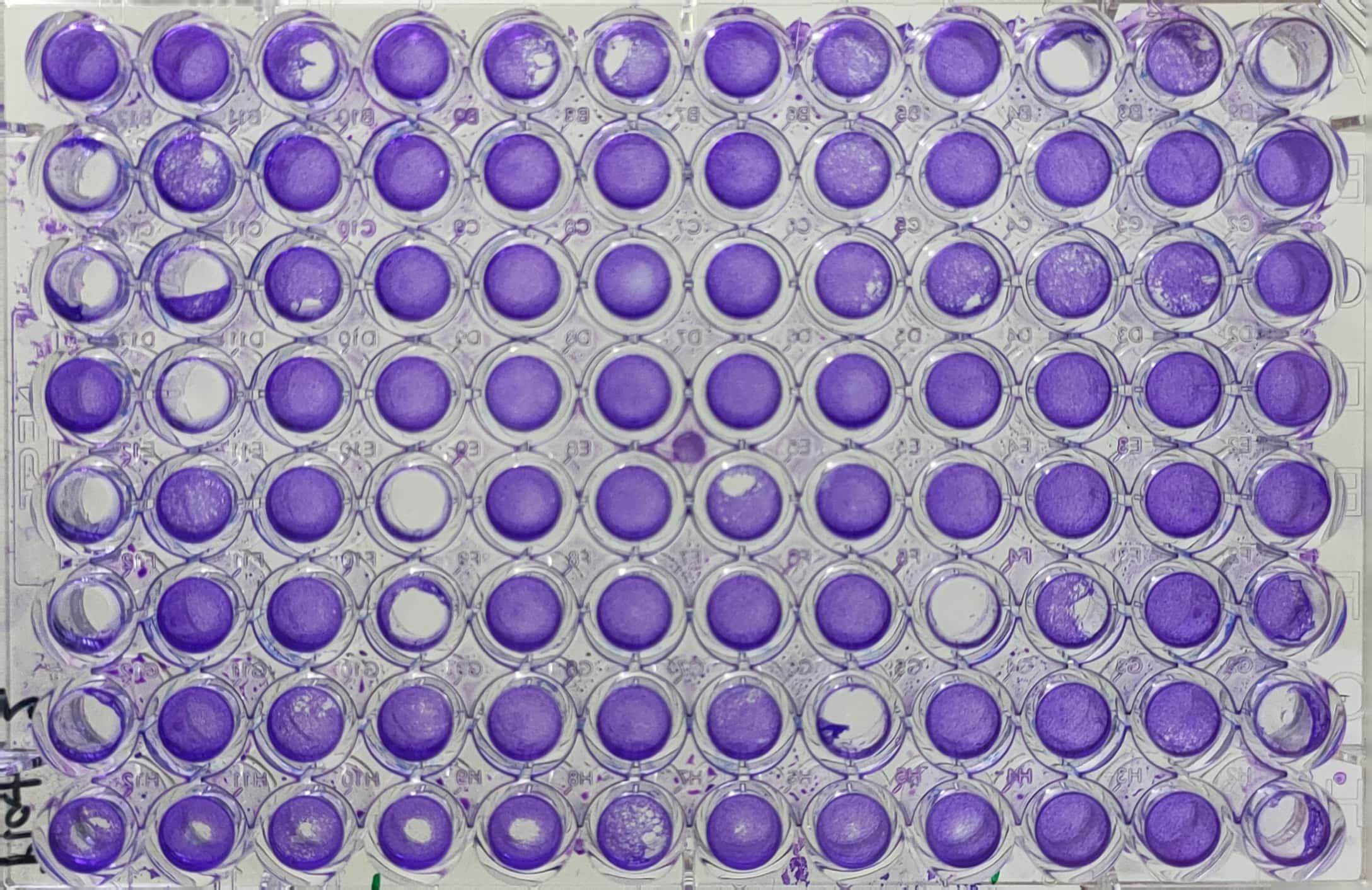 | 4/6 | 66.7 |
|  | 4 | 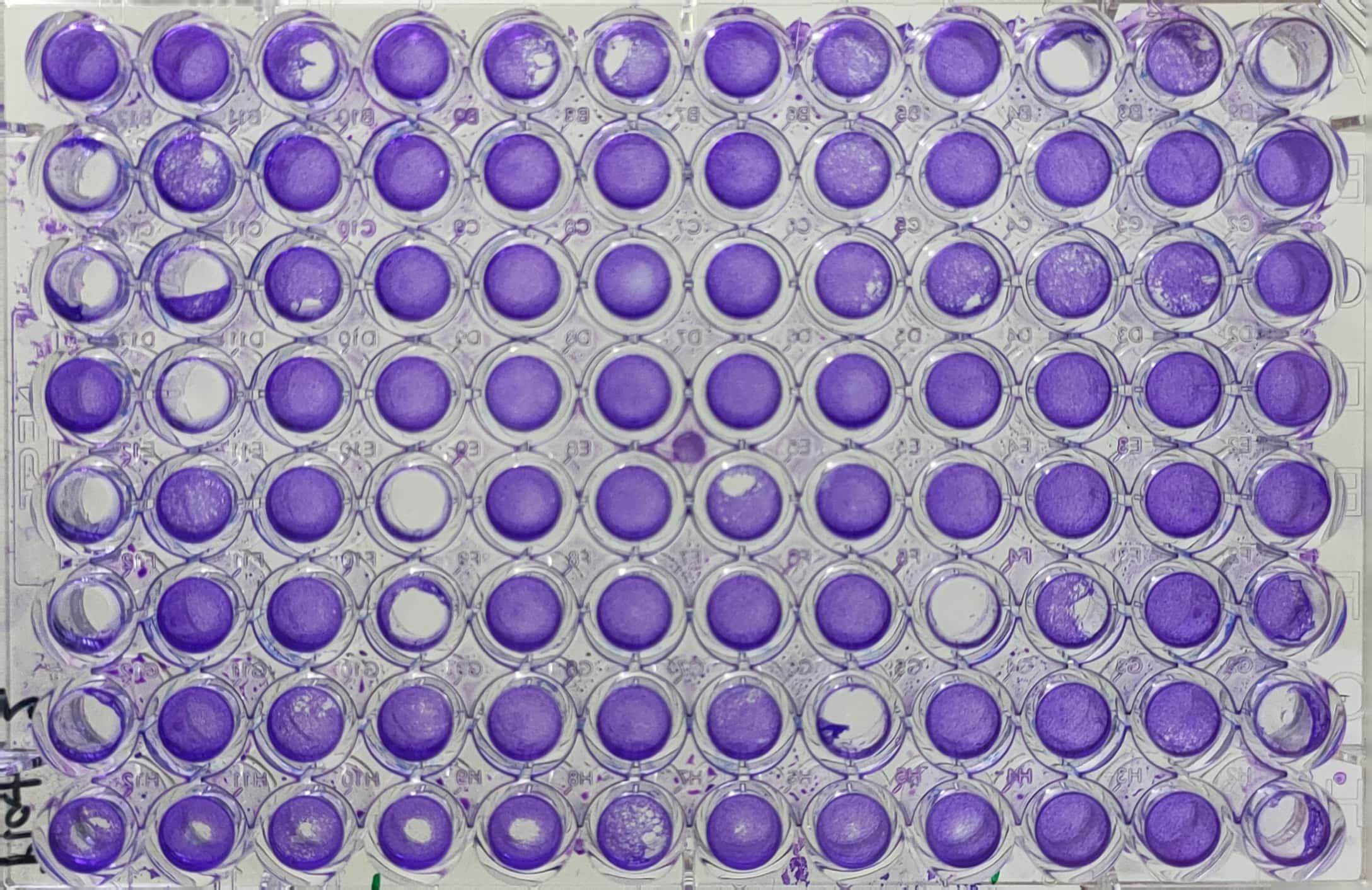 | 6/6 | 100 |
|  | 6 | 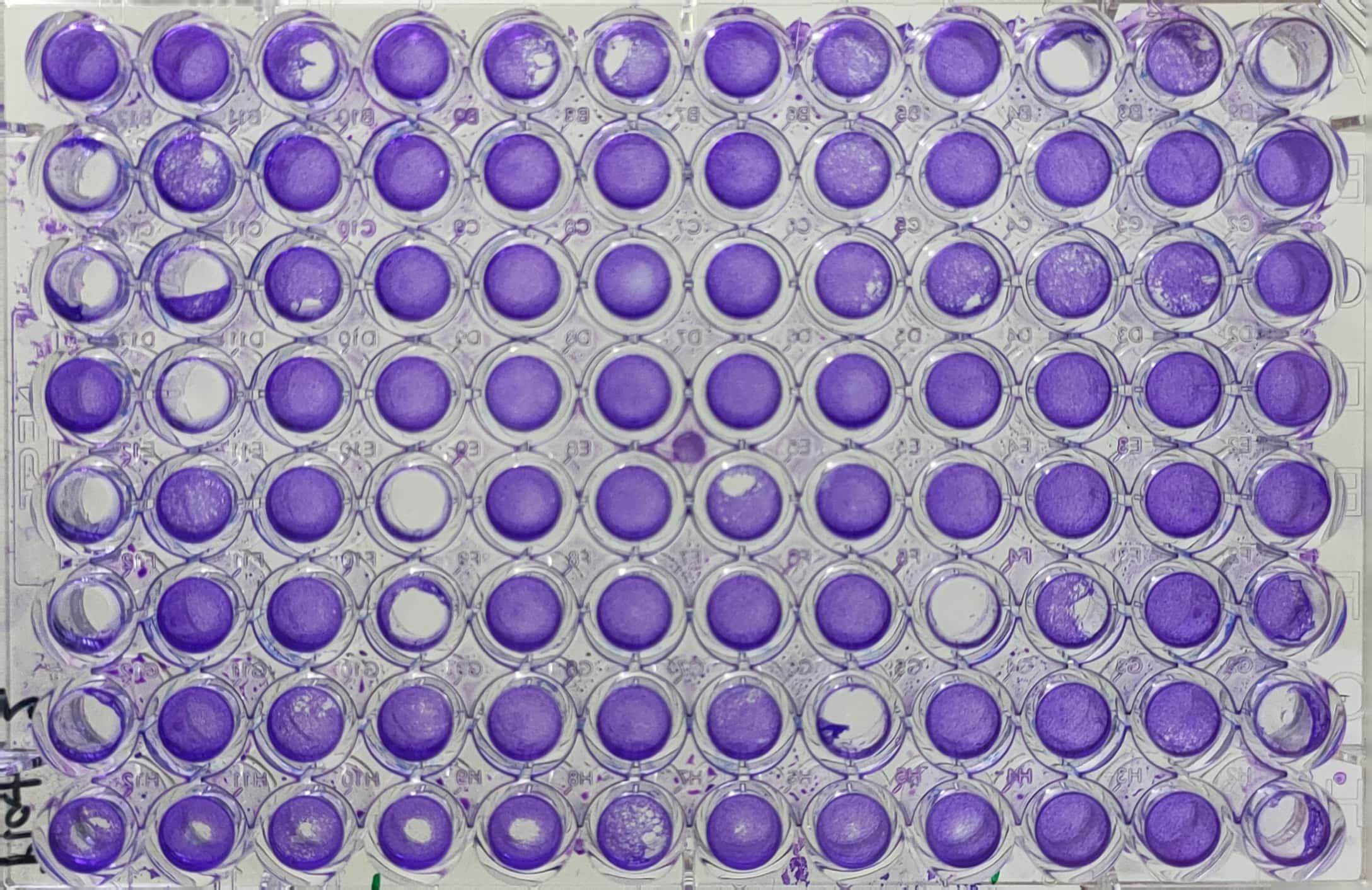 | 6/6 | 100 |
|  | 8 | 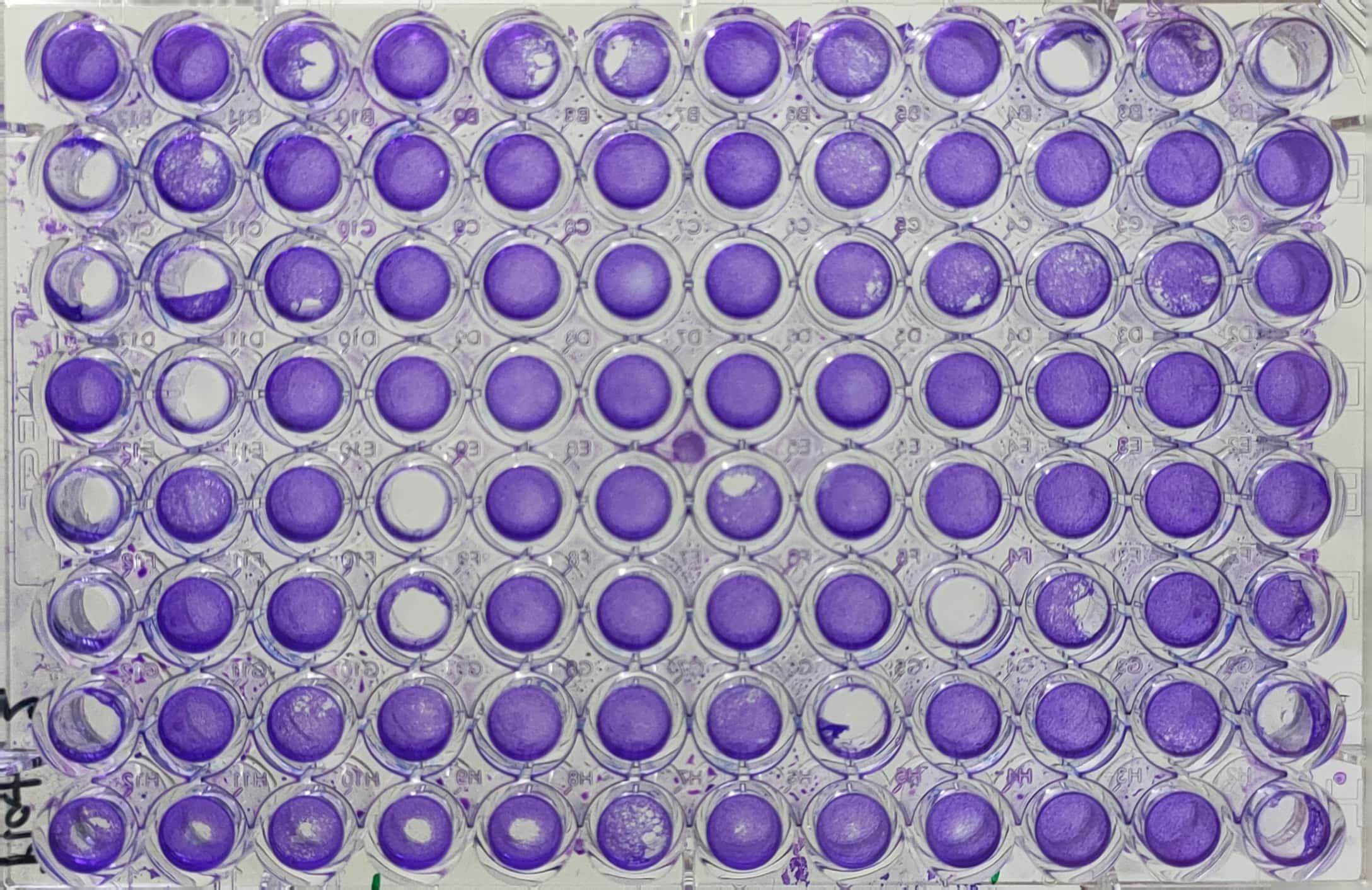 | 6/6 | 100 |
|  | 10 | 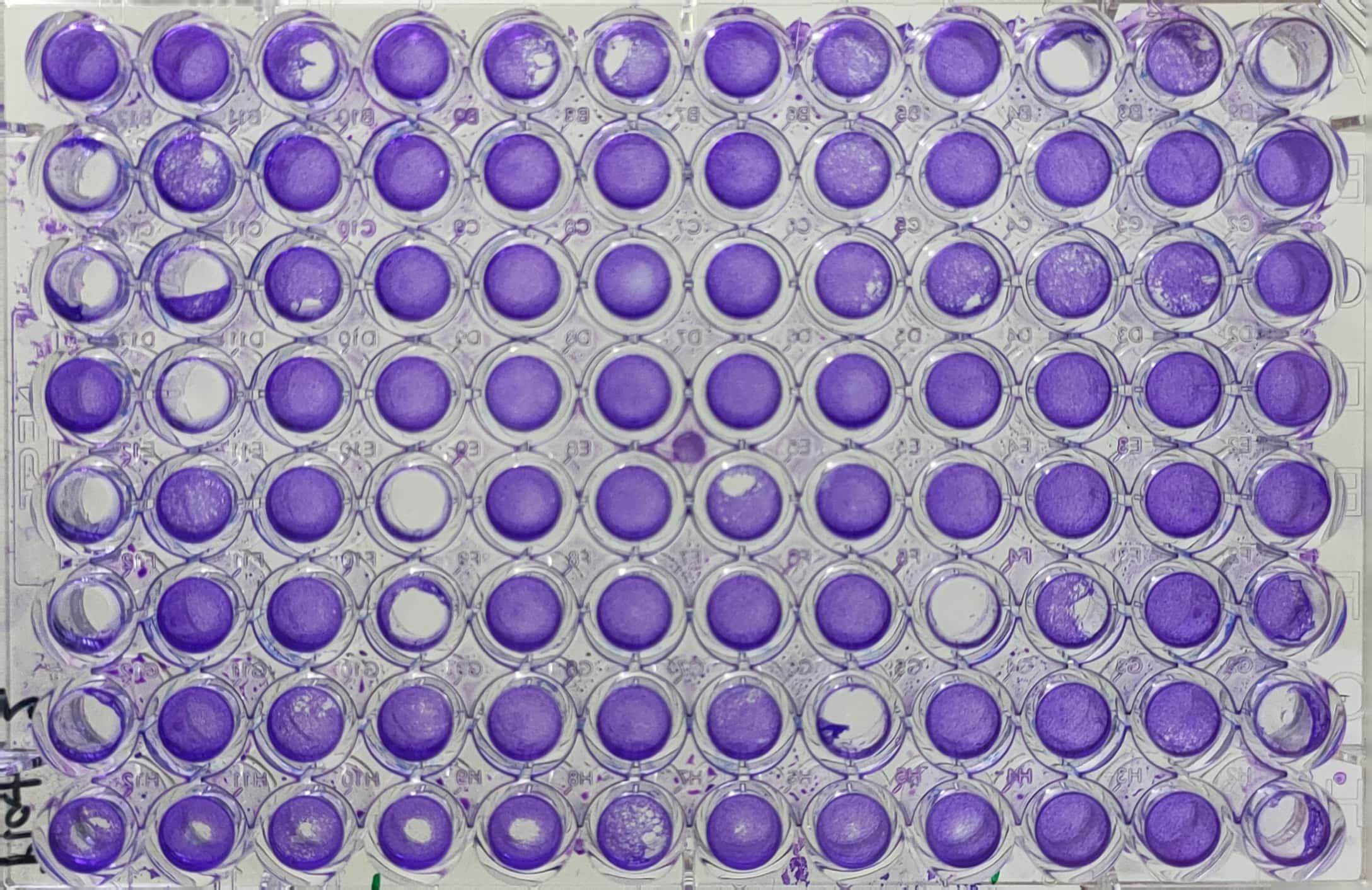 | 6/6 | 100 |

* 100% means 100% wells with no CPE observed.
